# Pilot study of a group clinical supervision model for medical students

**DOI:** 10.1177/10398562231186238

**Published:** 2023-06-24

**Authors:** Abby Moran, Amelia Shanahan, Alison Tomlin, Rowena Ivers, Susan J Thomas

**Affiliations:** Medical Student, Graduate School of Medicine, 8691 University of Wollongong, Wollongong, AU-NSW, Australia; Clinical Skills, Graduate School of Medicine, 8691 University of Wollongong, Wollongong, AU-NSW, Australia; Chair, Phase 3, Graduate School of Medicine, 8691 University of Wollongong, Wollongong, AU-NSW, Australia; Mental Health and Behavioural Science, Graduate School of Medicine, 8691 University of Wollongong, Wollongong, AU-NSW, Australia

**Keywords:** clinical supervision, burnout, medical students, reflection, professional development

## Abstract

**Objective:**

To trial a clinical supervision model with medical students, co-designed by students and clinicians, and evaluate its feasibility, acceptability, and perceived benefits.

**Method:**

Two clinical supervision groups, one online and one face-to-face, were conducted for six one-hour sessions, over 12 weeks. Clinical supervision was evaluated through mixed methods including attendance levels, focus groups, and quantitative surveys.

**Results:**

Thirteen students participated, including one rural and one regional group, each with a clinical supervisor. Attendance was 100%. Students viewed clinical supervision as a safe time for reflection on clinical experiences, validation from senior clinicians and peers, and connection to the medical community. Themes that emerged included strategies to prevent moral injury, self-care, and the need for a trusted clinical supervisor.

**Conclusion:**

The clinical supervision model received positive medical student evaluations and 100% attendance. This shows promise as an avenue to professionally support medical students as they navigate complex clinical training.

Clinical supervision has been defined as ‘the formal provision, by senior/qualified health practitioners, of relationship-based education and training that is case-focused and which manages, supports, develops, and evaluates the work of colleagues/supervisees’.^1^ It can be delivered in group or individual format. Clinical supervision is an integral part of training, support and professional development within nursing, allied health and psychiatry training. Across these professions, clinical supervision can reduce burnout, encourage professional growth, and promote reflection.^2^

Burnout is a condition associated with chronic stress characterised by emotional exhaustion, cynicism, and low professional efficacy.^3^ It is likened to the extinguishing of a candle that cannot continue burning brightly unless sufficient resources are being replenished.^4^ Medical school is a period of high stress that places students at significant risk of becoming burntout; contributing factors include heavy workload, limited support, hierarchical culture, and balancing the demands of study with one’s personal responsibilities.^5–7^ A recent global meta-analysis found one in two medical students to be currently experiencing burnout.^8^ These results are consistent with a national survey of Australian doctors and medical students.^6^

In nursing and allied health, effective clinical supervision reduces burnout and promotes staff retention.^9,10^ Opportunities to reflect with a more senior clinician have been shown to reduce rates of burnout and absenteeism.^11^ The term ‘supervision’ in the medical profession usually refers to the act of more senior doctors observing junior staff and medical students performing procedures and examinations. Clinical supervision, as defined by Milne^1^ is not routinely employed in the training of medical students. While clinical supervision has been evaluated for a number of health disciplines, there is limited literature regarding its potential benefits for medical students and junior doctors.

This pilot study was co-designed by medical students and clinical medical educators with the goal to evaluate the feasibility, acceptability, and utility of group clinical supervision in an Australian post-graduate medical school. Co-design involved a series of meetings between students and medical educators including medical practitioners and a clinical psychologist experienced in clinical supervision models to develop the model, student resources, and supervisor manual.

A clinical supervision model for medical students was developed based on clinical supervision models used in other health disciplines, including nursing and allied health while also drawing on the Balint model, which had been used previously with medical students and junior doctors.^12^ This medical school has previously attempted to establish Balint groups with expert facilitators; however, there was insufficient interest from medical students, perhaps due to the time commitment required. The new model involved sessions of groups of 4–10 students, held fortnightly with an experienced medical clinician as a supervisor.

Clinical supervision sessions followed a guided structure (Table 1), addressing themes contained in the Australian Medical Code of Conduct.^13^ The model was designed to address development of reflective practice while discussing clinical scenarios with the aim of reducing burnout and cynicism. This model utilised participative supervision, that is placed between authoritative supervision (a dyadic relationship between the supervisor and each of the supervisees), co-operative supervision (supervision by the group). A supervisor manual and student guide were developed to support delivery.

**Table 1.** Structure of group clinical supervision sessions

Duration	Content	Notes
10 minutes	Introductions and general check in	• Opening discussion
		• The initial session included information on clinical supervision, expectations of the group, and confidentiality
		• Limits of confidentiality in situations where follow-up was thought to be necessary, for example, if serious, unprofessional behaviour or mental health problems came to light in the group, the facilitator would need to refer the student to appropriate support or take other action as appropriate
		• Discussion of responsibilities of the supervisor and of those being supervised
10 minutes	Presentation of theme	Themes discussed included
		• providing good patient care, including ethical dilemmas
		• working with patients and their families, including with children and end of life care
		• working within a multidisciplinary team
		• working within the healthcare system
		• professionalism including good communication and navigating conflict
		• ensuring doctors’ health
30 minutes	Discussion of case	• If students do not have a relevant case, one can be provided by the facilitator
10 minutes	Closing	• Reiteration of theme, closing thoughts, final questions, or concerns

Groups met face-to-face or via teleconferencing software for an hour every 2 weeks, over 6 sessions. One of the investigation team who developed the model was a clinical psychologist experienced in use of clinical supervision model and the two clinical supervisors were experienced general practitioners and medical educators.

## Objectives

The objectives of this pilot study were to:

1. Trial a clinical supervision model with medical students.
2. Evaluate the feasibility, acceptability and perceived benefit to medical students through attendance levels, quantitative surveys and focus groups.

## Methods

Approximately 40 senior medical students (third–fourth year of a 4-year, graduate MD program) in two hubs, one regional and one rural, were invited to participate in the pilot project. Recruitment took place online through announcements on the university’s intranet page. Two clinical supervision groups each ran for six sessions from November 2021 to February 2022. During this phase of their studies, all students take part in a generalist year with training time split between general practice and emergency department settings. Recruitment took place online and the project was advertised on the university’s intranet page. Two clinical supervision groups each ran for six sessions from November 2021 to February 2022. A total of 13 students gave informed consent. The rural group was conducted in person and the regional group was online. The clinical supervision sessions were not recorded.

The feasibility and acceptability of clinical supervision sessions was assessed through attendance levels, surveys and focus groups. This study used a participatory action research framework where medical students participating in clinical supervision were also participating in the research process. Participatory action research is an established approach where the affected population are actively involved in research and reflection, which promotes empowerment, action, and sustainable change.^14^ Each group included a separate medical student participatory researcher. Focus groups were held after the third and sixth sessions. Focus groups were 1 hour long and included semi-structured, open-ended questions co-designed by the clinical academics and student researchers. Focus groups were facilitated and recorded by the two participatory action researchers/medical students. The supervisors were not present at the focus groups so that students could talk freely. The recordings used the teleconferencing software transcription and deidentified transcripts were reviewed by the researchers. Two investigators read and coded the transcripts using the NVivo software. Focus group qualitative data were analysed using thematic analysis.

An evaluation form consisted of questions with Likert scale responses to assess students’ satisfaction with the group. Evaluation results were compared between groups. The Maslach Burnout Inventory (MBI) was administered via an anonymous online Qualtrics survey before the first and after the sixth supervision sessions to assess changes in burnout. Fisher’s exact test was used for comparisons, due to the small sample size to compare the results of the MBI burnout domains pre and post clinical supervision.

Ethics approval was obtained from the university human research ethics committee and participants gave informed consent.

## Results

Thirteen students enrolled in the study (nine females, four males, mean age 29.70), with nine in one regional hub and four in a rural location. Gender proportions were similar to the overall MD cohort (60.24% female; 39.76% male). Attendance was 100% across both groups for the six sessions.

Nine participants from one site took part in the focus groups. Twenty-eight categories emerged from the focus group transcripts, which were clustered into core themes (Table 2). Students from both sites rated the program highly in their final evaluation forms (Table 3). There were no significant differences between groups in satisfaction levels (*p* > 0.05 in all cases).

**Table 2.** Qualitative themes and examples

*Theme*	*Example quote*
*Burnout*	*‘I feel like I’ve run a bit of a marathon and I am puffed out and need to sit on the bench and have a bit of a breather. Still enjoy medicine of course but just need a little break to re-energise at this point’ [M1].*
Students reported high levels of burnout and emotional exhaustion throughout the study. Overall responses indicated burnout affecting attendance and engagement in being linked to increased cynicism	
*Student Wellbeing*	*‘…this is the only time during this program where we asked each other how we are actually doing. ... I don’t think that anyone else really asks that from like a faculty perspective...’ [F4]* *‘Sometimes it feels like you’re on a bit of an island in medicine... it is nice to have this dedicated time to talk through issues. And it seems like we all pretty much have experienced similar things in some way or another. So that’s quite nice and therapeutic just to hear other people’s experiences too’. [M1]* *“I don’t think in medical school we really have any sort of time or space for... students to all connect about our experiences, whether they’re reflecting on clinical cases, or kind of doing anything else, it’s just that opportunity for connection that’s lacking and [Group Clinical Supervision] provides that’ [F4].* *‘...having your feelings validated, or you’re having your exhaustion validated, or feeling like I’m not being dramatic, I’m not making this up’ [F2].*
All students supported the continuation of group clinical supervision as an intervention to support medical students during their training. The placed high value on the sharing of clinical experiences, connection with other students, and the validation and support provided by a senior clinical supervisor	
*Consolidation of learning*	*‘The benefits of this sort of group is that you can delve deeper into the things that you may not talk about in passing, with your colleague in the hallway because it’s just no time it’s more sort of superficial things and you probably don’t want to burden them with your issues and that sort of thing so it’s nice to have this formal space where you have time to discuss some heavy issues with your colleagues and also the fact that it is with your colleagues or peers is beneficial compared to talking to your partner or your friend or someone who’s not necessarily been through experiences’ [M1].* *‘[Group Clinical Supervision] has given me a really healthy way to reflect on different situations, and not necessarily answer finding, or anything like that, but just, I guess, boundaries, in a way, a way to discuss things. And as F2 said, you know, perhaps not even through discussing a certain clinical situation that applied specifically to you, hearing about other people’s experiences, for me has been reflective’ [F3].*
Participants identified that group clinical supervision encouraged reflection on clinical practice and consolidation of clinical skills; they emphasised the value of self-reflection and debriefing throughout medical school education	
*Improve Clinical Competence*	*‘… it’s been like, I guess associated with me being a better practitioner and just, I guess being more reflective, generally happier knowing that I’m sharing the experience of many other students and I think it’s something that I personally really wish that we had from Phase One’ [F3].* *‘[Group Clinical Supervision] is really useful because the other people in this group weren’t necessarily in [the] situation and so [there] is less... judgment, and emotional baggage, I feel like it’s a safer space... I find it more beneficial... sharing my experiences and also, I think I find it less burdensome … to actually listen to others debriefing … from an emotional perspective I’m someone that tends to carry a lot of emotions, and so I feel like listening to people’s debrief is almost therapeutic and a way for me to learn but not even such a burden in some way there’s no so many like deep intense attached emotions that there often is at the time of events’[F3].*
Participants identified that group clinical supervision encouraged reflection on clinical practice and consolidation of clinical skills; they emphasised the value of self-reflection and debriefing throughout medical school education	

*Note*: M = male; F = female.

**Table 3.** Results of post-intervention, anonymous online survey of student satisfaction with the group clinical supervision program, comparing the rural and regional groups

How would you rate this program overall?	5-Excellent	4	3-Satisfactory	2	1	0-Unsatisfactory
Rural	1 (33)	2 (66)	0	0	0	0
Regional	5 (63)	3 (38)	0	0	0	0
How likely are you to recommend this program to other students?	5-Very likely	4-Somewhat likely	3-Neither likely nor unlikely	2-Somewhat unlikely	1-Extremely unlikely	0
Rural	2 (66)	1 (33)	0	0	0	0
Regional	5 (63)	3 (38)	0	0	0	0
This program met my expectations	Yes	No	—	—	—	—
Rural	3 (100)	0	—	—	—	—
Regional	8 (100)	0	—	—	—	—
This program was useful towards my degree	5-Extremely useful	4-Very useful	3-Moderately useful	2-Slightly useful	1-Not at all useful	0
Rural	2 (66)	0	1 (33)	0	0	0
Regional	5 (63)	2 (25)	1 (13)	0	0	0
How likely are you to do this program again	5-Very likely	4-Somewhat likely	3-Neither likely nor unlikely	2-Somewhat unlikely	1-Extremely unlikely	0
Rural	2 (66)	1 (33)	0	0	0	0
Regional	7 (88)	1 (12)	0	0	0	0

*Note*: Numbers of students endorsing each response are indicated, with percentages in brackets. The regional group was online and the rural group was face-to-face. Fisher’s exact test indicated no significant differences in evaluations of the groups (*p* > 0.05 in all cases).

All 13 participants completed the MBI before and after participation in group clinical supervision. The results of the MBI showed overall reductions in proportions of those with high degrees of burnout (Table 4); however, Fisher’s exact test did not yield statistically significant results (*p* > 0.05).

**Table 4.** Maslach burnout inventory results pre and post clinical supervision

**Pre-clinical supervision (*n* = 13)**			**Post-clinical supervision (*n* = 11)**	
**Emotional exhaustion**	**Number of participants in score range**	**Percentage**	**Number of participants in score range**	**Percentage**
High (>30)	6	46	2	18
Moderate (18–29)	5	38	7	64
Low (<17)	2	15	2	18
Mean score (SD)	25.23 (8.92)		23.55 (6.49)	
Depersonalisation				
High (>12)	4	31	2	18
Moderate (6–11)	3	23	4	36
Low (<5)	6	46	5	45
Mean (SD)	7.3 (5.16)		6.55 (4.14)	
Personal accomplishment				
High (>40)	4	31	4	36
Moderate (34–39)	4	31	2	18
Low (<33)	5	38	5	45
Mean (SD)	29.31 (7.58)		35.18 (7.47)	

## Discussion

This pilot study indicates that clinical supervision is acceptable to students in a graduate medical program. Students gave strongly positive evaluations of the clinical supervision program, which was further supported by 100% attendance for all six sessions across both sites. As hypothesised, clinical supervision was seen as a safe space for students to connect and to share their experiences, providing a supportive approach to personal and professional development.^15^

Participating medical students reported high levels of burnout, low enthusiasm and poor engagement in clinical placement. This is consistent with observations that burnout and cynicism in the medical workforce commence early in training.^6,7^ Overall, there were reduced numbers of students endorsing high levels of burnout after completing the supervision pilot. These were not statistically significant. Larger and longer term studies are needed to understand effects on burnout, taking into account the timing of exams and assessments. Furthermore, students reported high periods of stress as a result of lack of connection to other students, and limited opportunities to share their clinical experiences and reflect on their current practice. Qualitative focus group analyses indicate that clinical supervision addressed this need.

A limitation of the study is the very small sample size. Nonetheless, this pilot study supports the need for further research investigating the impact of clinical supervision in medical training. Given previous research supporting the positive impact of clinical supervision on stress levels, professional development, and accountability in other health professions, further studies should be considered for medical students and junior doctors.^16^

Clinical supervision has potential to be both feasible and acceptable for use within the medical school curriculum, with potential for use with junior doctors. This is a small pilot study involving qualitative analysis, in a self-selected cohort of students, and more research is required to further assess true effect on burnout.
